# Immunosuppressive Ability of *Trichinella spiralis* Adults Can Ameliorate Type 2 Inflammation in a Murine Allergy Model

**DOI:** 10.1093/infdis/jiad518

**Published:** 2023-11-28

**Authors:** Wenjie Shi, Qinwei Xu, Yan Liu, Zhili Hao, Yue Liang, Isabelle Vallée, Xihuo You, Mingyuan Liu, Xiaolei Liu, Ning Xu

**Affiliations:** State Key Laboratory for Diagnosis and Treatment of Severe Zoonotic Infectious Diseases, Key Laboratory for Zoonosis Research of the Ministry of Education, Institute of Zoonosis, and College of Veterinary Medicine, Jilin University, Changchun; Department of Pulmonary and Critical Care Medicine, Qilu Hospital of Shandong University, Qingdao; College of Public Health, Jilin Medical University, China; State Key Laboratory for Diagnosis and Treatment of Severe Zoonotic Infectious Diseases, Key Laboratory for Zoonosis Research of the Ministry of Education, Institute of Zoonosis, and College of Veterinary Medicine, Jilin University, Changchun; State Key Laboratory for Diagnosis and Treatment of Severe Zoonotic Infectious Diseases, Key Laboratory for Zoonosis Research of the Ministry of Education, Institute of Zoonosis, and College of Veterinary Medicine, Jilin University, Changchun; Unité Mixte de Recherche Biologie moléculaire et Immunologie Parasitaire, Anses, Institut national de recherche pour l'agriculture, l'alimentation et l'environnement, Laboratoire de Santé Animale, Ecole Nationale Vétérinaire d’Alfort, Maisons-Alfort, France; Beijing Agrichina Pharmaceutical Co, Ltd, Beijing, China; State Key Laboratory for Diagnosis and Treatment of Severe Zoonotic Infectious Diseases, Key Laboratory for Zoonosis Research of the Ministry of Education, Institute of Zoonosis, and College of Veterinary Medicine, Jilin University, Changchun; State Key Laboratory for Diagnosis and Treatment of Severe Zoonotic Infectious Diseases, Key Laboratory for Zoonosis Research of the Ministry of Education, Institute of Zoonosis, and College of Veterinary Medicine, Jilin University, Changchun; State Key Laboratory for Diagnosis and Treatment of Severe Zoonotic Infectious Diseases, Key Laboratory for Zoonosis Research of the Ministry of Education, Institute of Zoonosis, and College of Veterinary Medicine, Jilin University, Changchun

**Keywords:** allergic asthma, alternatively activated macrophages, immunosuppressive response, ovalbumin, *Trichinella spiralis*

## Abstract

**Background:**

There is an increase in the global incidence of allergies. The hygiene hypothesis and the old friend hypothesis reveal that helminths are associated with the prevalence of allergic diseases. The therapeutic potential of *Trichinella spiralis* is recognized; however, the stage at which it exerts its immunomodulatory effect is unclear.

**Methods:**

We evaluated the differentiation of bone marrow–derived macrophages stimulated with *T spiralis* excretory-secretory products. Based on an ovalbumin-induced murine model, *T spiralis* was introduced during 3 allergy phases. Cytokine levels and immune cell subsets in the lung, spleen, and peritoneal cavity were assessed.

**Results:**

We found that *T spiralis* infection reduced lung inflammation, increased anti-inflammatory cytokines, and decreased Th2 cytokines and alarms. Recruitment of eosinophils, CD11b^+^ dendritic cells, and interstitial macrophages to the lung was significantly suppressed, whereas Treg cells and alternatively activated macrophages increased in *T spiralis* infection groups vs the ovalbumin group. Notably, when *T spiralis* was infected prior to ovalbumin challenge, intestinal adults promoted proportions of CD103^+^ dendritic cells and alveolar macrophages.

**Conclusions:**

*T spiralis* strongly suppressed type 2 inflammation, and adults maintained lung immune homeostasis.

## Teaser

*Trichinella spiralis* adults maintain lung immune homeostasis by increasing the proportion of alveolar macrophages and CD103^+^ dendritic cells while reducing the recruitment of interstitial macrophages. They promote the development of Treg cells and alternatively activated macrophages to alleviate allergic inflammation.

## Graphical Abstract

**Graphical Abstract:**
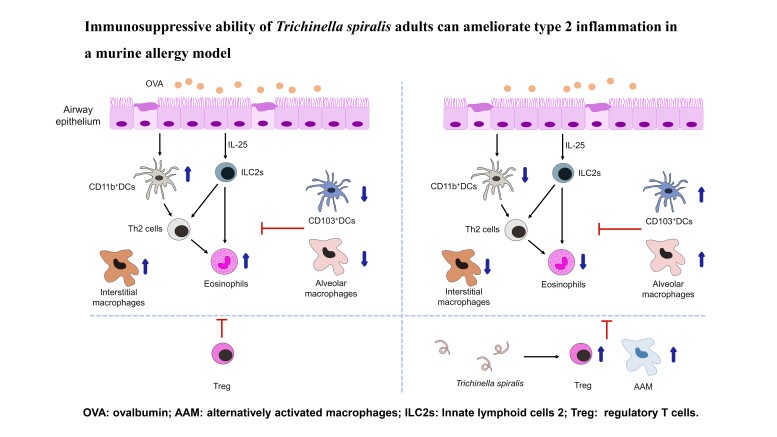

Allergies, typical immune-mediated inflammatory diseases, are on the rise globally [1]. The immunoregulatory potential of helminths has substantial therapeutic potential in immune-mediated inflammatory diseases, such as inflammatory bowel diseases and allergies [2–5]. However, the relationship between the Th2-type immune response and the immunosuppressive response induced by helminths such as *Trichinella spiralis* makes it challenging to discern their immunoregulatory effects on inflammatory diseases. *T spiralis* has a unique life cycle, completing parasitism within the same host across various stages—intestinal, migration, and muscular. Infective muscular larvae (ML) undergo 4 molts at 31 hours postinfection [6], developing into adults that reach sexual maturity and fertilization within 3 days in the intestinal tract [7]. Female adults produce newborn larvae (NBL) after 5 days that eventually parasitize muscle cells via the circulatory system [8]. Adult worms in the intestines are eventually eliminated [9].

The innate and adaptive immune systems of the host resist *T spiralis* through killing, weeping (intestinal mucus secretion), and sweeping (enhanced intestinal peristalsis) mechanisms. Macrophages and neutrophils contribute to killing *T spiralis* [10], whereas innate lymphoid cells 2, Th2 cells, and intestinal epithelial cells induce a strong type 2 immune response to eliminate *T spiralis* from the intestine [11]. However, *T spiralis* has developed a sophisticated immune evasion mechanism for long-term coexistence with hosts [12]. Because of the unique life cycle of *T spiralis*, the effects of its 3 life stages on the host immune response are different. Furthermore, the 3 stages of *T spiralis* cannot be entirely discerned, making it difficult to distinguish the immunomodulatory effects of different developmental stages. The ownership of the regulatory capacity of Th2-type immune responses and immunosuppressive responses remains elusive.

We investigated the regulatory effect of excretory-secretory products (ESPs) at different stages on bone marrow–derived macrophages (BMDMs) in vitro [13, 14]. Based on the immunoregulation of ESPs, our study examined the anti-inflammatory effect and immunoregulatory ownership of adults at 3 stages of allergic inflammation: the sensitization, effector, and treatment stages. We assessed changes in immune cells and cytokines in an ovalbumin (OVA)–induced model. The inhibitory effect of adults on allergic inflammation was stronger in the sensitization and effector phases than in the treatment phase. Our findings suggest that *T spiralis* adults induce an immunosuppressive response to reduce OVA-induced type 2 inflammation.

## METHODS

### Animals and Parasites

Female C56BL/6 mice and Wistar rats aged 6 to 8 weeks were procured from the Jilin University Experimental Animal Center. All animal experiments adhered to the guidelines for the care and use of laboratory animals [15] and were approved by the Ethical Committee of Jilin University, affiliated with the Provincial Animal Health Committee (ethical clearance KT202202140). *T spiralis* (ISS534) was obtained from the serial passage of rats in our laboratory.

### Asthma Model and Infection With *T spiralis*

Mice were initially sensitized with 20-μg OVA and 50-μL Imject Alum adjuvant (Thermo Fisher Scientific) in a 100-μL volume on days 0, 7, and 14. Control groups consisted of mice administered with either saline or alum adjuvant. Mice were subsequently exposed to aerosolized 1% OVA for 30 minutes via the airway on days 28, 29, and 30. The mice were humanely euthanized on day 37. In alignment with the infection timing, the group infected with *T spiralis* larvae was stratified into 4 subgroups corresponding to days −3, 15, 25, and 31.

### Culture and Induction of BMDMs

Bone marrow cells were obtained from the isolated leg bones. The cells were treated with red cell lysate, cultured in RPMI-1640 medium supplemented with 10% fetal bovine serum and 25 ng/mL of recombinant murine macrophage colony-stimulating factor, and incubated in an environment at 37 °C and with 5% CO_2_. The same medium was replaced 2 days later, and ESPs (at 20- and 50-μg concentrations) were added for 24 hours on day 4. Mature BMDMs were collected on day 5 for flow cytometry analysis, and the supernatant was collected to assess cytokine levels.

### Statistical Analysis

All data were analyzed with Prism software (version 5; GraphPad), and the results are presented as mean and SD. To assess differences among groups, 1- and 2-way analyses of variance were employed. Significance levels were denoted as *P < .05, **P < .01, and ***P < .001.

## RESULTS

### Differentiation of BMDMs Induced by ESPs of *T spiralis*

**Figure 1.**
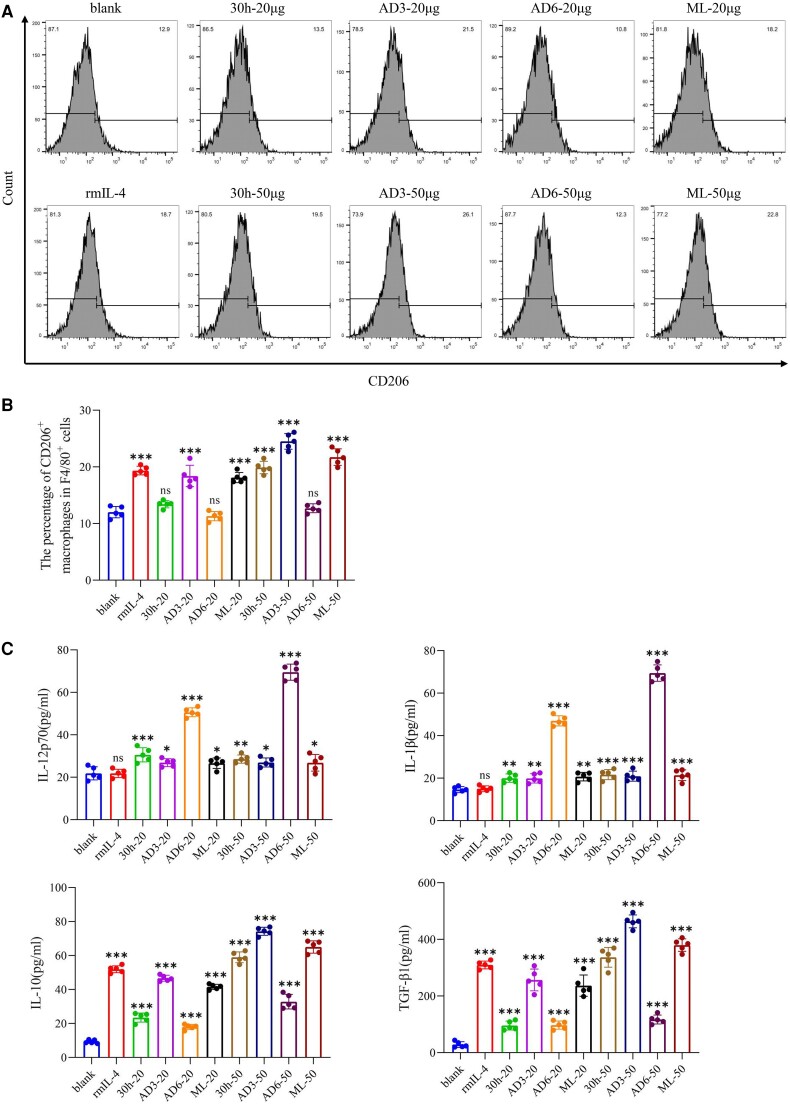
Immunoregulation of BMDMs by ESPs at various *Trichinella spiralis* stages. *A*, BMDMs were exposed to 20/50 μg of ESPs, and flow cytometry analysis revealed CD206^+^ macrophage levels. *B*, Proportion of CD206^+^ macrophages. *C*, Concentrations of interleukins 1β, 10, and 12p70 (IL-1β, IL-10, and IL-12p70) and transforming growth factor β1 (TGF-β1) in the cell culture supernatant. Data represent the mean ± SD from 5 independent experiments. ns, not significant. *P < .05. **P < .01. ***P < .001. One-way analysis of variance, followed by a Dunnett multiple-comparisons test. BMDM, bone marrow–derived macrophage; ESP, excretory-secretory product.

The proportion of F4/80^+^ BMDMs exceeded 98% (Supplementary Figure 1*A*), indicating the high purity and reactivity of the BMDMs. Stimulation with rmIL-4 resulted in an increased proportion of CD206^+^ macrophages (Figure 1*A* and 1*B*), confirming the reactivity of BMDMs. When stimulated with 20 μg of ESPs, the CD206 expression was upregulated by ESPs (AD3 and ML), whereas ESPs (30 hours and AD6) did not exhibit the same effect. As the dose of ESPs increased to 50 μg, CD206 expression was consistently upregulated, except in the AD6 group. The cytokine profiles secreted by BMDMs varied in response to different ESPs. All ESPs led to upregulated levels of interleukins 12p70 and 1β (IL-12p70 and IL-1β), with the AD6 group (20 and 50 μg) showing particularly high levels. Additionally, ESPs induced elevated levels of anti-inflammatory cytokines (interleukin 10 [IL-10] and transforming growth factor β1 [TGF-β1]), particularly in the 3 groups (30 hours, AD3, and ML; Figure 1*C*). Among these, ESPs (AD3) induced the highest levels of anti-inflammatory cytokines, consistent with CD206 expression, indicating that ESPs (AD3) possess a strong ability to induce the alternative activation of macrophages.

### *T spiralis* Adults Alleviate Allergic Inflammation in the Lungs

**Figure 2.**
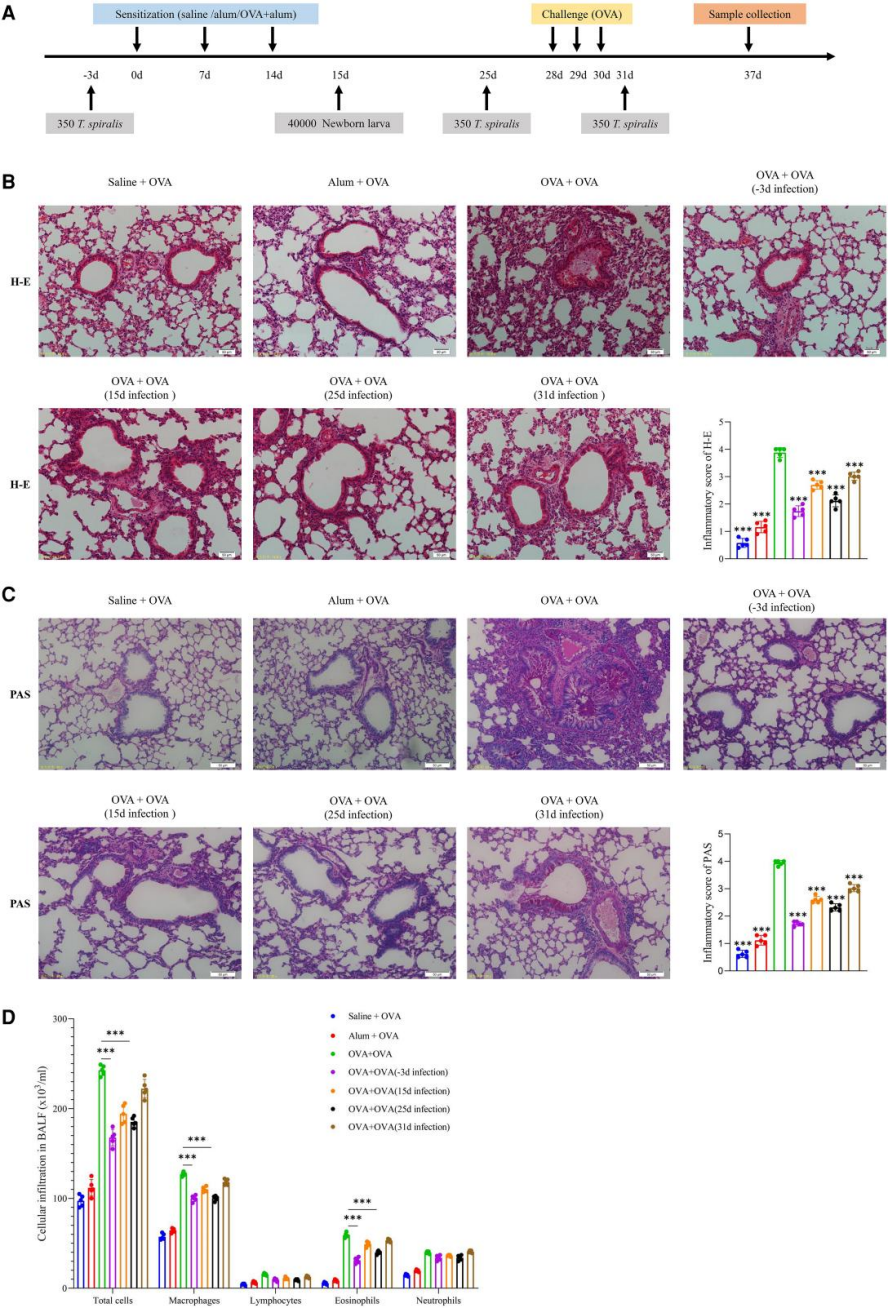
*Trichinella spiralis* mitigates allergic inflammation in the lungs. *A*, Experimental design. Mice underwent sensitization with OVA/alum via intraperitoneal injection on days 0, 7, and 14, followed by aerosolized OVA challenge on days 28, 29, and 30. A total of 350 muscular larvae were orally infected on days −3, 25, and 31, whereas 40 000 newborn larvae were introduced via tail vein injection on day 15. Mice were euthanized and analyzed on day 37. *B*, Lung sections were prepared and stained with hematoxylin and eosin (H-E). Inflammatory cell infiltration and bronchiolar hyperplasia were semiquantitatively assessed on a scale from absent (0) to minimal (1), mild (2), moderate (3), marked (4), and massive (5). *C*, Lung sections were stained with periodic acid–Schiff (PAS), and mucus production and goblet cell hyperplasia were also evaluated. *D*, Total cells, macrophages, lymphocytes, eosinophils, and neutrophils in BALF were stained with DiffQuik and analyzed. Data are expressed as the mean ± SD from 5 independent experiments. ***P < .001. *B* and *C*, One-way analysis of variance, followed by a Dunnett multiple-comparisons test. *D*, Two-way analysis of variance, followed by a Dunnett multiple-comparisons test. BALF, bronchoalveolar lavage fluid; OVA, ovalbumin.

Considering the regulatory effect of ESPs on macrophages, we artificially infected mice with *T spiralis* and observed immunoregulatory effects at 3 stages: sensitization (day −3 and 15 of infection), challenge (day 25 of infection), and treatment (day −3; Figure 2*A*). The infection efficiency of NBL was determined by quantifying the ML (Supplementary Figure 2). The OVA sensitization and challenge group (OVA group) exhibited more inflammatory cell infiltration, mucus production, and bronchiolar wall proliferation than the saline and adjuvant control groups (Figure 2*B* and 2*C*). In the *T spiralis* infection group at day −3, the ML presence affected the subsequent OVA challenge stage. Therefore, mice were infected with NBL via the tail vein on day 15 as a control. Adults significantly reduced inflammatory cell infiltration, goblet cell metaplasia, and mucus production in lung tissue during the sensitization, challenge, and treatment stages. NBL infection was less effective in suppressing allergic inflammation than adult infection at day −3. Cell counts in bronchoalveolar lavage fluid (BALF) included various immune cells, such as macrophages, neutrophils, eosinophils, and lymphocytes. The results showed that adults reduced total cell counts and macrophage and eosinophil infiltration induced by OVA, particularly on days −3 and 25 of the infection groups (Figure 2*D*).

### *T spiralis* Adults Reduce OVA-Specific Immunoglobulin

**Figure 3.**
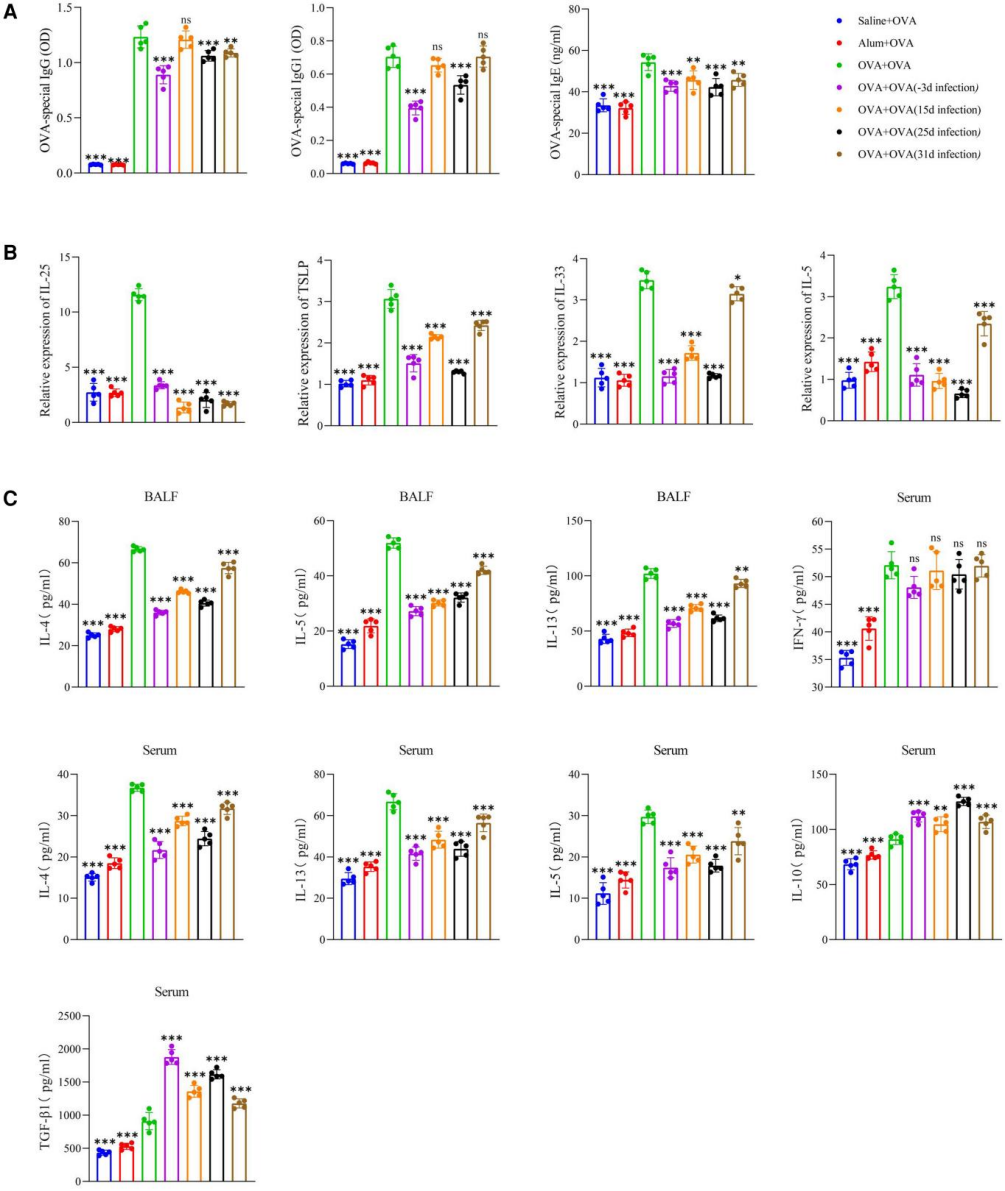
Regulation of OVA-specific antibodies and inflammatory cytokines by *Trichinella spiralis* adults. *A*, Serum levels of OVA-specific IgG, IgG1, and IgE. *B*, Lung mRNA expression of IL-5, IL-25, IL-33, and TSLP assessed via real-time quantitative polymerase chain reaction. *C*, Levels of inflammatory-related cytokines in BALF or serum. Data are expressed as the mean ± SD from 5 independent experiments. ns, not significant. *P < .05. **P < .01. ***P < .001. One-way analysis of variance, followed by a Dunnett multiple-comparisons test. BALF, bronchoalveolar lavage fluid; IFN-γ, interferon γ; Ig, immunoglobulin; IL, interleukin; OD, optical density; OVA, ovalbumin; TGF-β1, transforming growth factor β1; TSLP, thymic stromal lymphopoietin.

We evaluated the effect of *T spiralis* on OVA-specific immunoglobulin in serum (Figure 3*A*). OVA–immunoglobulin G (IgG) and OVA–immunoglobulin G1 (IgG1) levels were significantly decreased in the *T spiralis* infection group at day −3 but not in the NBL infection group at day 15. Furthermore, the levels of OVA-IgG and OVA-IgG1 were significantly reduced in the *T spiralis* infection group at day 25. Moreover, *T spiralis* infection reduced the level of OVA–immunoglobulin E (IgE), particularly in the *T spiralis* infection groups at days −3 and 25.

### Anti-inflammatory Cytokines Induced by *T spiralis* Adults Suppress Type 2 Inflammation

To further evaluate the immunomodulatory ability of *T spiralis* adults, we examined anti- and proinflammatory cytokine levels. In lung tissues, *T spiralis* significantly reduced the mRNA expression of thymic stromal lymphopoietin (TSLP) and interleukins 5, 25, and 33 (IL-5, IL-25, and IL-33; Figure 3*B*). In particular, *T spiralis* infection on day 31 had mildly affected the expression of IL-5, TSLP, and IL-33 as compared with the other treatments. OVA significantly promoted the levels of Th2 cytokines (interleukin 4 [IL-4], IL-5, and interleukin 13 [IL-13]) in BALF (Figure 3*C*). Furthermore, *T spiralis* infection lowered IL-4, IL-5, and IL-13 levels in BALF, with the *T spiralis* infection group at day −3 exhibiting lower cytokine levels than the *T spiralis* infection group at day 15. According to cytokine changes in the BALF, the *T spiralis* infection groups showed decreased levels of IL-4, IL-5, and IL-13 in serum, whereas interferon γ levels were not significantly altered. In contrast, the *T spiralis* infection upregulated IL-10 and TGF-β1 levels. Generally, the *T spiralis* infection group at day −3 exhibited the highest level of TGF-β1, and the *T spiralis* infection group at day 25 had the highest level of IL-10 among the 4 groups.

### *T spiralis* Adults Affect Lung Immune Cells in OVA-Induced Allergic Models

**Figure 4.**
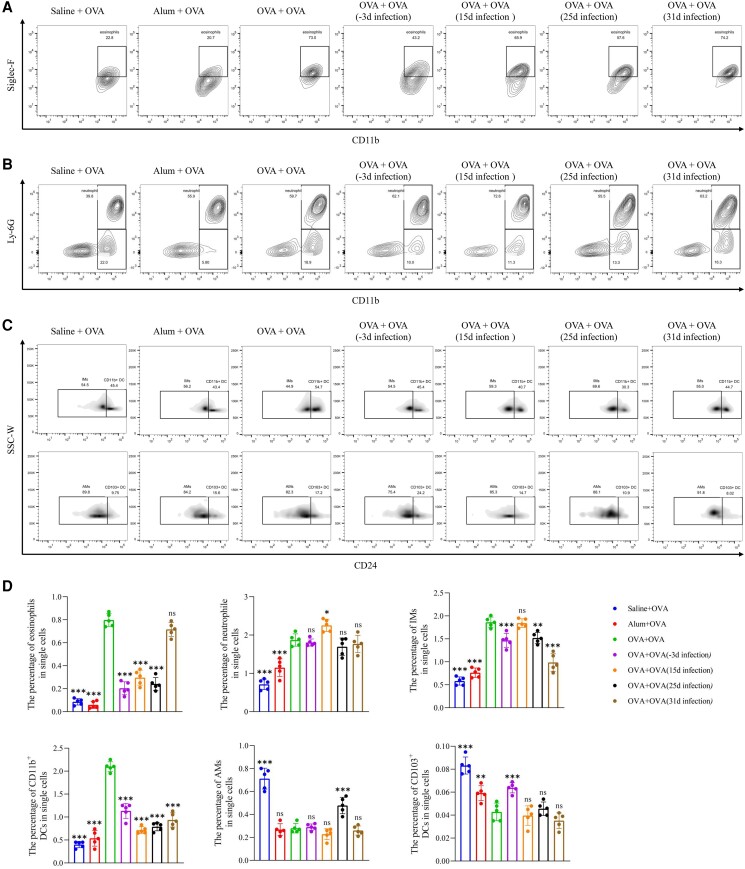
The immunoregulation of immune cells in the lung by *Trichinella spiralis* adults. *A* and *B*, Representative dot plots of Siglec-F^+^ CD11b^+^ eosinophils and Ly-6G^+^ CD11b^+^ neutrophils in the lungs. *C*, Representative dot plots of IMs, AMs, CD103^+^ DCs, and CD11b^+^ DCs in the lungs. *D*, Proportions of eosinophils, neutrophils, IMs, AMs, CD103^+^ DCs, and CD11b^+^ DCs in single cells. Data are expressed as the mean ± SD from 5 independent experiments. ns, not significant. *P < .05. **P < .01. ***P < .001. One-way analysis of variance, followed by a Dunnett multiple-comparisons test. AM, alveolar macrophage; DC, dendritic cell; IM, interstitial macrophage; OVA, ovalbumin; SSC-W, side scatter width.

We conducted flow cytometry analysis to assess changes in eosinophils, neutrophils, CD103^+^ dendritic cells (DCs), CD11b^+^ DCs, alveolar macrophages (AMs), and interstitial macrophages (IMs) in the mouse lung (Supplementary Figure 3*A* and 3*B*). The OVA group exhibited higher proportions of eosinophils (CD11c^−^, CD11b^+^, and SiglecF^+^) and neutrophils (CD11c^−^, CD11b^+^, and Ly6G^+^) as compared with the control groups (Figure 4*A* and 4*D*). The proportion of eosinophils decreased in the *T spiralis* infection groups at days −3, 15, and 25 but did not change significantly in the day 31 group vs the OVA group. However, the proportion of neutrophils increased only in the *T spiralis* infection group on day 15 when compared with the OVA group (Figure 4*B* and 4*D*). Previous studies have categorized lung macrophages into 2 subsets: AMs (MHCII^+^, CD11c^hi^, CD11b^int^, and CD24^−^) and IMs (MHCII^+^, CD11b^hi^, and CD24^−^) [16]. Sensitization with alum or OVA significantly reduced the proportion of AMs while increasing the proportion of IMs (Figure 4*C* and 4*D*). The proportions of IMs were significantly lower in the *T spiralis* infection groups at days −3, 15, and 25 vs the OVA group. Yet, only the group infected with *T spiralis* at day 25 exhibited a higher proportion of AMs than the OVA group. In lung tissues, CD103^+^ DCs (MHCII^+^, CD11c^hi^, CD11b^+^, and CD24^+^) and CD11b^+^ DCs (MHCII^+^, CD11b^hi^, and CD24^+^) are the primary antigen-presenting cells that induce T-cell activation. The results demonstrated that OVA sensitization decreased the proportion of CD103^+^ DCs while increasing the proportion of CD11b^+^ DCs. Only *T spiralis* infection on day −3 promoted the proportion of CD103^+^ DCs as compared with the OVA group. In contrast, *T spiralis* infection significantly reduced the proportion of CD11b^+^ DCs.

**Figure 5.**
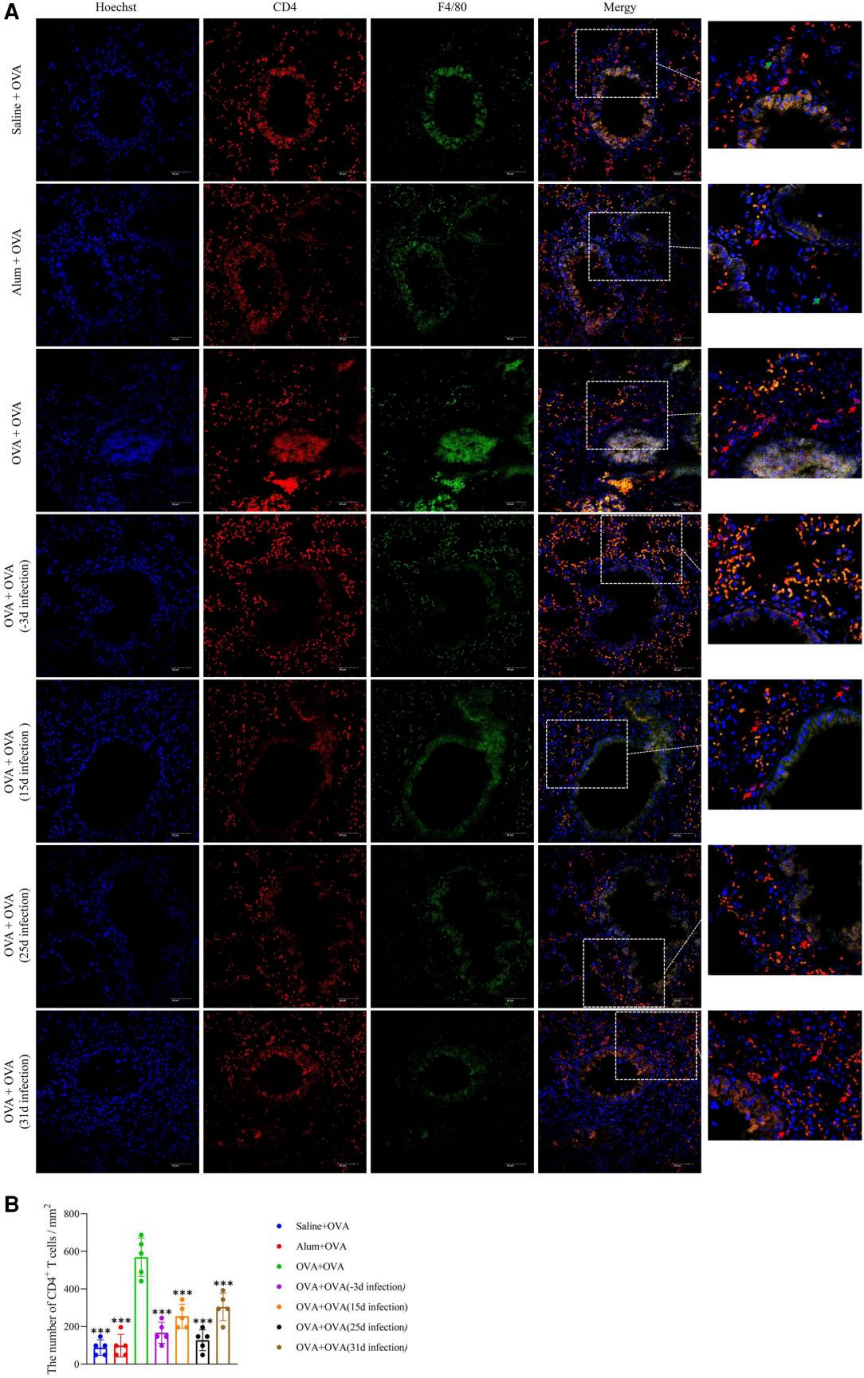
Immunofluorescence of CD4^+^ T cells and macrophages in lung. *A*, Immunostained lung sections show F4/80, CD4, and Hoechst. CD4^+^ T cells and F4/80^+^ macrophages are indicated by arrows. *B*, The number of CD4^+^ T cells per square millimeter was counted. Data are expressed as the mean ± SD from 5 independent experiments. ***P < .001. One-way analysis of variance, followed by a Dunnett multiple-comparisons test. OVA, ovalbumin.

To further investigate the involvement of T cells and macrophages in lung tissues, we employed anti-CD4 and anti-F4/80 antibodies for cell labeling. *T spiralis* infection mitigated cell infiltration and decreased the number of T cells, particularly in the infection groups on days −3 and 25 as compared with the OVA group. However, no significant change in the number of macrophages was observed (Figure 5).

### *T spiralis* Adults Affect Lymphocytes of the Spleen and Peritoneal Macrophages

**Figure 6.**
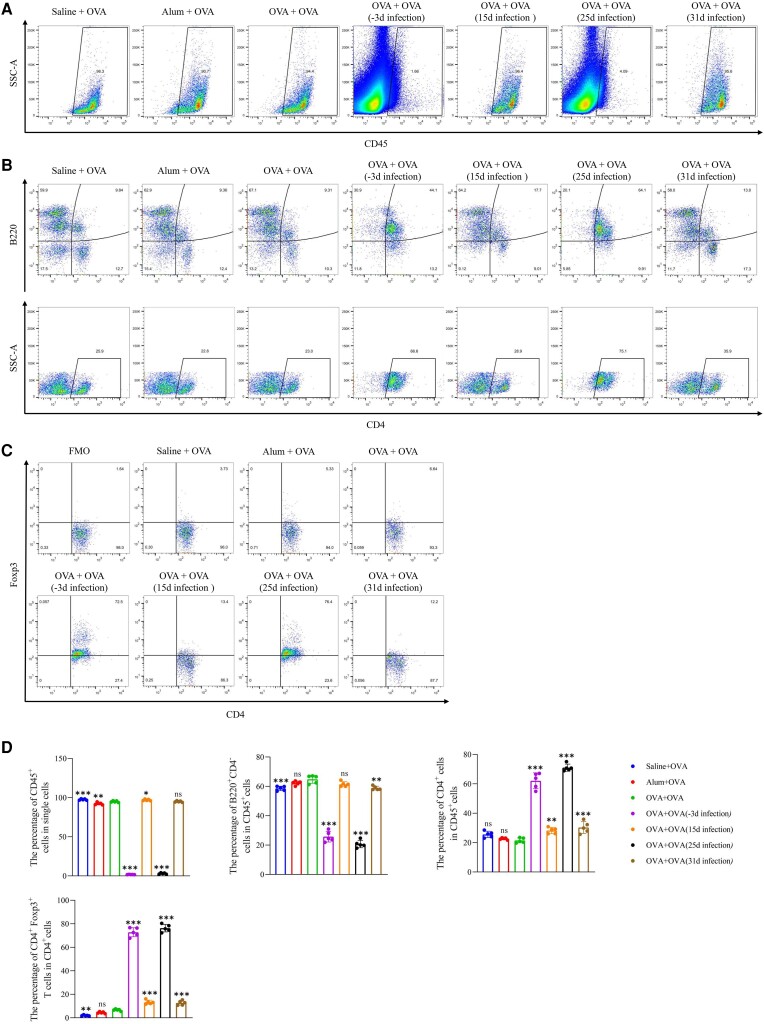
Suppression of immune cell proliferation and promotion of Treg cell differentiation by *Trichinella spiralis* adults in the spleen. Flow cytometry analysis of (*A*) CD45^+^ immune cells, (*B*) B220^+^ B cells and CD4^+^ T cells, and (*C*) Foxp3^+^ Treg cells in the spleen. *D*, Proportion of CD45^+^ immune cells in single cells, B cells and CD4^+^ T cells in CD45^+^ cells, and Treg cells in CD4^+^ T cells. Data are expressed as the mean ± SD from 5 independent experiments. ns, not significant. *P < .05. **P < .01. ***P < .001. One-way analysis of variance, followed by a Dunnett multiple-comparisons test. OVA, ovalbumin; SSC-A, side scatter area.

**Figure 7.**
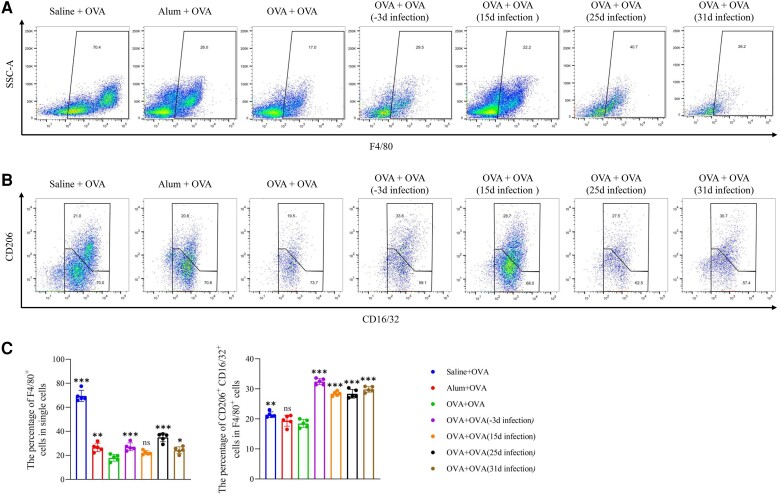
Induction of alternative activation in peritoneal macrophages by *Trichinella spiralis* adults. *A* and *B*, Representative dot plots of F4/80^+^ macrophages and CD16/32^+^ CD206^+^ cells. *C*, Proportion of F4/80^+^ macrophages in single cells and CD16/32^+^ CD206^+^ cells in F4/80^+^ macrophages. Data are expressed as the mean ± SD from 5 independent experiments. ns, not significant. *P < .05. **P < .01. ***P < .001. One-way analysis of variance, followed by a Dunnett multiple-comparisons test. OVA, ovalbumin; SSC-A, side scatter area.

To evaluate the effect of lymphocytes and macrophages, we detected the differentiation of splenocyte subsets and peritoneal macrophages (Supplementary Figure 1*B* and 1*C*). *T spiralis* infection on days −3 and 25 significantly reduced the proportion of CD45^+^ cells in the spleen (Figure 6*A* and 6*D*). Examination of the changes in B- and T-cell subsets in OVA-induced allergic mice postinfection showed a higher number of B cells (CD45^+^, B220^+^, and CD4^−^) in the OVA group than the saline group. However, infection with *T spiralis* on days −3 and 25 significantly decreased the proportion of B cells (Figure 6*B* and 6*D*). Conversely, *T spiralis* infection increased the proportion of CD4^+^ T cells (CD45^+^ and CD4^+^), particularly on days −3 and 25. The proportion of Treg cells (CD45^+^, CD4^+^, and FoxP3^+^) in the *T spiralis* infection groups was significantly higher than that in the OVA group, particularly on days −3 and 25 (Figure 6*C* and 6*D*). Furthermore, peritoneal macrophages are reported to induce an immunosuppressive response during the intestinal phase of *T spiralis* infection [17]. Significantly fewer F4/80^+^ macrophages were observed in the OVA group than the saline group, indicating that alum/OVA sensitization affected the proliferation of peritoneal macrophages (Figure 7*A* and 7*C*). Although *T spiralis* infection increased the proportion of macrophages, this change was not statistically significant in the NBL infection group at day 15. Similarly, *T spiralis* infection induced a higher proportion of CD16/32^+^ and CD206^+^ macrophages than the OVA control group (Figure 7*B* and 7*C*). Overall, these findings illustrate that *T spiralis* effectively modulated the host's immune response to suppress inflammation.

## DISCUSSION

This study revealed the capacity of early-stage ESPs to induce alternative activation of macrophages, characterized by an upregulated CD206 expression and the production of anti-inflammatory cytokines, such as IL-10 and TGF-β1. Among the time points studied, ESPs from AD3 exhibited the strongest induction capacity, possibly related to their role in helping NBL evade the host's immune response. Furthermore, ESPs, being complex heterologous antigens, triggered an immune response [18], including an increase in proinflammatory cytokine levels such as IL-12p70 and IL-1β. Therefore, we focused on understanding the immunosuppressive capacity of early *T spiralis* infection stages in the context of allergic diseases.

We examined the immunomodulatory effects of the migration and muscular stages during the challenge stage by injecting NBL into the tail vein. Using NBL infection as a control, we evaluated the immunoregulatory effect of intestinal adults on allergic inflammation during the sensitization stage. Our findings confirmed that adults alleviated the infiltration of inflammatory cells, reduced mucus production, and inhibited bronchiolar wall proliferation. Additionally, adults significantly lowered the levels of OVA-specific antibodies (IgG, IgG1, and IgE) during the sensitization and challenge stages. B cells were pivotal in antibody class switching, as influenced by various cytokines, including IL-4, which regulates class switching to IgE [19–21]. The reduction in OVA-specific IgE levels was associated with the suppression of the type 2 immune response and a decrease in IL-4 levels. Interestingly, significant changes in OVA-specific IgG and IgG1 levels were observed in the groups infected on days −3 and 25 vs the OVA group, paralleling changes in B-cell populations in the spleen. This suggests that adults possess the ability to regulate humoral immunity. This study demonstrated that *T spiralis* reduces the mRNA expression levels of alarm cytokines (IL-25, IL-33, and TSLP), which are crucial in inducing the type 2 innate immune response. Furthermore, it resulted in decreased levels of Th2 cytokines (IL-4, IL-5, and IL-13) after infection, while interferon γ levels remained largely unchanged. In contrast, *T spiralis* induced high levels of anti-inflammatory cytokines (IL-10 and TGF-β1). Therefore, we concluded that the inhibitory effect of *T spiralis* on type 2 inflammation primarily relies on immunosuppression rather than a Th1-type immune response.

The host's immune environment during the 3 allergy stages exhibited variations, and these different survival conditions faced by *T spiralis* in the 4 groups led to diverse immunomodulatory capacities of the worms. To comprehend the immunoregulatory effects on lung tissues, it was imperative to investigate changes in various immune cells within the lung. Although it is debated whether neutrophils contribute to the type 2 immune response, the elevated levels of interleukins 6, 8, and 17 in the lungs drove neutrophils into the tissue, potentially amplifying inflammation [22, 23]. In our study, the increased presence of neutrophils in the lungs induced by alum/OVA is likely associated with the development of inflammation. Interestingly, NBL infection before the challenge stage led to an increased influx of neutrophils into the lungs, possibly linked to the circulation of NBL through lung tissue. Additionally, eosinophils are crucial in allergic inflammation [24]. Cytokines such as IL-5 and granulocyte-macrophage colony-stimulating factor are critical in eosinophil differentiation and maturation. Activated eosinophils present processed antigens to T cells, stimulating cell proliferation, and release various proinflammatory substances that amplify the inflammatory response, such as IL-4, IL-5, and IL-13 and major basic proteins 1 and 2 [25, 26]. This study demonstrated that *T spiralis* infection prior to the challenge stage reduced the recruitment of eosinophils, which is important in limiting inflammation and promoting tissue homeostasis. Furthermore, *T spiralis* reduced the number of infiltrating CD4^+^ T cells around the bronchioles.

Lung macrophages can be categorized into 2 populations—AMs and IMs. AMs located in the alveolar space are exposed to the external environment and serve to eliminate potentially harmful substances [27]. These AMs are derived from embryonic progenitors and have the capacity for self-proliferation under lung homeostasis [28]. During the challenge phase of allergy, AMs upregulate YM1 and Arg1 expression, inducing an immunosuppressive response to limit the lung DC function and antigen-specific antibody production [29–31]. We found a significant decrease in the AM proportion in the OVA group as compared with the saline group. The AM proportion was promoted by adults only before the challenge phase. Additionally, IMs within the lung interstitium are maintained by circulating monocytes and are considered to prevent immune responses against harmless inhaled antigens [32]. There is considerable debate regarding which subsets of IMs promote or suppress inflammation [30, 33, 34]. Our findings revealed that OVA induced an increase in the proportion of IMs in mouse lungs, contrasting with the trend observed for AMs. Adults significantly reduced the IM population during the allergy development, while NBL had no obvious effect, indicating that the inhibition of IM recruitment depended on the immunoregulation of adults.

Th2 cells are pivotal in aggravating the inflammatory response through the production of cytokines such as IL-4, IL-5, and IL-13 [35]. DCs are crucial for initiating the adaptive immune response because of their robust migration and antigen presentation capabilities [36]. In the lungs, DCs are typically divided into 3 subsets at a steady state: plasmacytoid DCs and conventional DCs (comprising CD11b^+^ cDCs and CD103^+^ DCs). Under inflammatory conditions, monocyte-derived DCs are recruited into lung tissues and produce proinflammatory chemokines to attract eosinophils and effector T cells in the airways. Monocyte-derived DCs are similar to CD11b^+^ cDCs, which participate in and exacerbate allergic inflammation [37, 38]. Consequently, we investigated these 2 subsets as CD11b^+^ DCs. CD11b^+^ DCs are reported to prime the type 2 adaptive immune response, whereas CD103^+^ DCs promote the type 1 adaptive immune response, helping to suppress excessive type 2 inflammation through the release of IL-12 [39]. In our study, the number of CD11b^+^ DCs increased in the lungs following OVA stimulation, while the proportion of CD103^+^ DCs decreased. *T spiralis* infection significantly reduced the proportion of CD11b^+^ DCs, which was associated with a subsequent weakening of the adaptive type 2 immune response. Interestingly, the proportion of CD103^+^ DCs increased significantly when *T spiralis* infection occurred before the sensitization stage. The immunosuppressive environment induced by adult worms reduced proinflammatory cell subsets in the lung. Additionally, adults demonstrated the ability to maintain immune homeostasis by promoting the differentiation of AMs and CD103^+^ DCs in the lungs. However, this immunomodulatory capacity was effective only before OVA challenge.

Helminths create an immunosuppressive microenvironment to survive within parasitic tissues. Additionally, helminths can suppress inflammation in other tissues, primarily relying on anti-inflammatory cytokines [5, 40]. Anti-inflammatory cytokines are primarily produced by Treg cells and alternatively activated macrophages [5, 17, 41, 42]. CD4^+^ Foxp3^+^ Treg cells induced by *T spiralis* possess a stronger ability to migrate and suppress the immune response than normal Treg cells [43]. According to flow cytometry analysis, adult worms significantly reduced the CD45^+^ immune cell population in the spleen before the onset of allergic inflammation. In contrast, *T spiralis* infection after the challenge phase had no effect on CD45^+^ immune cells, and NBL infection increased the proportion of CD45^+^ immune cells. Therefore, we concluded that immunoregulation by intestinal adults is crucial in suppressing lung inflammation before allergic inflammation is activated. Consistent with the changes observed in OVA-specific IgG and IgG1, adults reduced the proportion of B220^+^ B cells before allergic inflammation was activated. Conversely, *T spiralis* infection increased the proportion of Treg cells. In the *T spiralis* infection groups on days −3 and 25, 70% of CD4^+^ T cells were Treg cells, indicating a significant reduction in T helper cells. Thus, Treg cells induced by adults are vital in suppressing inflammation. Furthermore, during the intestinal phase of *T spiralis* infection, the immune microenvironment in the peritoneal cavity is significant in anti-inflammation [44, 45]. Macrophages constitute a significant proportion of the peritoneal cavity's immune cells, with alternatively activated macrophages being primarily responsible for inducing an anti-inflammatory response [17]. Our results demonstrated that the use of alum/OVA disrupted immune homeostasis in the peritoneal cavity, leading to a decreased proportion of macrophages. Adults had the ability to restore the proportion of macrophages, whereas NBL infection did not. Additionally, *T spiralis* infection upregulated CD206 expression, suggesting an increase in the proportion of alternatively activated macrophages.

In conclusion, *T spiralis* adults induce an immunosuppressive response through the differentiation of alternatively activated macrophages and Treg cells, as well as by increasing the levels of IL-10 and TGF-β1. This immunosuppression effectively suppresses excessive type 2 immune responses. Moreover, before the onset of allergic inflammation, adults contribute to maintaining the balance of immune cells in the lung and protecting pulmonary immune homeostasis. To conclude, this study elucidated the immunoregulatory effects of *T spiralis* adults in inhibiting the development of allergic diseases. It provides insights into how the immunoregulatory capabilities of these worms can be harnessed for potential treatments of allergic diseases, such as through infection with single-sex *T spiralis* or the utilization of adult-derived proteins.

## Supplementary Data

Supplementary materials are available at *The Journal of Infectious Diseases* online (http://jid.oxfordjournals.org/). Supplementary materials consist of data provided by the author that are published to benefit the reader. The posted materials are not copyedited. The contents of all supplementary data are the sole responsibility of the authors. Questions or messages regarding errors should be addressed to the author.

## Supplementary Material

jiad518_Supplementary_Data

## Notes

***Acknowledgments.*** We thank Lin Li for her help in experiment.

***Author contributions.*** N. X., X. L., W. S., and Q. X. contributed to conceive and design this study. W. S., N. X., Z. H., and Y. Liang acquired the data. W. S., N. X., Y. Liu, I. V., X. Y., M. L., and Q. X. did the analysis and interpretation of data. N. X., X. L., and M. L. provided the funding for this study. All authors contributed to drafting the manuscript and revising it critically for important intellectual content.

***Date availability.*** All relevant data are within the article and its supplementary materials.

***Financial support*.** This work was supported by the National Key Research and Development Program of China (2021YFC2600202); the National Natural Science Foundation of China (32202835, 32230104); the Science and Technology Talents and Platform Plan of Yunnan Province (Academician and Expert Workstation, 202305AF150167) and the Fundamental Research Funds for the Central Universities. Funding to pay the Open Access publication charges for this article was provided by the National Key Research and Development Program of China (2021YFC2600202).
