# Supplementary material for: Immunosuppressive Ability of Trichinella spiralis Adults Can Ameliorate Type 2 Inflammation in a Murine Allergy Model
Source: J Infect Dis. 2023 Nov 28;229(4):1215–28. doi: 10.1093/infdis/jiad518 (PMC11011206; doi:10.1093/infdis/jiad518)
Supplement: jiad518_Supplementary_Data [file jiad518_supplementary_data.docx]

Immunosuppressive ability of Trichinella spiralis adults can ameliorate type 2 inflammation in a murine allergy model

**Running title:** T. spiralis adults ameliorate type 2 inflammation

Wenjie Shi^1, #^, Qinwei Xu^2, #^, Yan Liu^3^, Zhili Hao^1^, Yue Liang^1^, Isabelle Vallée^4^, Xihuo You^5^, Mingyuan Liu^1^, Xiaolei Liu^1, $^, Ning Xu^1, *^

^1^ State Key Laboratory for Diagnosis and Treatment of Severe Zoonotic Infectious Diseases, Key Laboratory for Zoonosis Research of the Ministry of Education, Institute of Zoonosis, and College of Veterinary Medicine, Jilin University, Changchun 130062, China

^2^ Department of Pulmonary and Critical Care Medicine, Qilu Hospital of Shandong University (Qingdao), Qingdao266035, China

^3^ College of public health, Jilin Medical University, Jilin, Jilin, China

^4^ UMR BIPAR, Anses, INRAE, Ecole Nationale Vétérinaire d’Alfort, Laboratoire de Santé Animale, Maisons-Alfort, France

^5^ Beijing Agrichina Pharmaceutical Co., Ltd, Wangzhuang Industrial Park, Airport Road, Shahe, Changping District, Beijing, China

^#^ These authors contributed equally to this work.

^*^ Corresponding author: Ning Xu, PhD, Institute of Zoonosis, Jilin University, 5333 Xi’an Road, Changchun, Jilin 130062, China ([beyond44141@163.com](mailto:beyond44141@163.com)).

^$^ Alternate corresponding author: Xiaolei Liu, PhD, Institute of Zoonosis, Jilin University, 5333 Xi’an Road, Changchun, Jilin 130062, China ([liuxlei@163.com](mailto:liuxlei@163.com)).

1. **Materials and methods**
   1. **ESPs of *Trichinella spiralis* at Different Periods**

Worms were collected at 30 hours (30 h), 3days (AD3), 6 days (AD6) and 35 days (ML) according to the reported method. Next, worms were washed three times with saline containing 100 U penicillin/mL and 100 μg streptomycin/mL. The collected ML were cultured with pig bile salts (2.4 mg/mL in saline, Solarbio life sciences, Beijing, China) at 37 °C in 5% CO_2_ for 2 h. The worms were cultured for 18 hours in RAMI 1640 at 37 °C and 5% CO_2_. The culture supernatant was collected and centrifuged with an Amicon Ultra3 Centrifugal Filter Unit at 4 °C and 8000 rpm/min. The obtained ESPs were quantified by BCA kit (Solarbio life sciences, Beijing, China) and stored at -80 °C.

- 1. **Analysis of Cells in Bronchoalveolar Lavage Fluid (BALF)**

Mice were euthanized on Day 37, and BALF was obtained by repeatedly flushing the lungs from the trachea with 1 mL PBS. Cells were obtained by centrifugation at 3000 rpm/min for10 min and stained with DiffQuik (Solarbio life sciences, Beijing, China) according to the manufacturer’s instructions. Two hundred cells were randomly selected and classified under a light microscope.

- 1. **Histology of the Lungs**

The lung tissues were obtained and fixed in formalin for 24 h. The lung tissues were paraffin-embedded and sectioned. Sections were dewaxed and stained with hematoxylin and eosin (H&E) or periodic acid-Schiff (PAS). The stained sections were scanned under a microscope, and the pathological changes were scored according to the scoring system from absent (0), minimal (1), moderate (3), to marked (4).

- 1. **Real-time PCR**

Total RNA was extracted from the lungs. cDNA was synthesized by reverse transcription of RNA and used to determine IL-25, IL-33, IL-5 and TSLP gene expression with a PerfectStart Uni RT & qPCR Kit (TransGen Biotech, Beijing, China). The transcript abundance of genes was analyzed with the ΔΔct method. The primer sequences are shown in Table S1.

- 1. **Analysis of Cytokines and Ovalbumin-specific Immunoglobulin**

The concentrations of IL-4, IL-5, IL-13, IL-10, IFN-γ, and TGF-β1 in serum or BALF were detected with ELISA kits (Mei mian, Jiangsu, China) according to the manufacturer’s instructions. Similarly, the level of OVA-specific IgE was detected with an ELISA kit (Mei mian, Jiangsu, China). The results were measured at 450 nm with an ELISA plate reader (BioTek).

Levels of OVA-specific IgG and IgG1 were detected by indirect ELISA. 96-wells were coated with 100 μL OVA (5 μg/mL) overnight at 4 ℃. The serum was diluted (1:500) and incubated for 1 h at 37 ℃. HRP-anti-mouse IgG1 and HRP-goat anti-mouse IgG (Abcam, Cambridge, United Kingdom) were incubated for 1 h at 37 ℃. TMB was added and incubated for 10 min to obtain results at 450 nm with an ELISA plate reader (BioTek).

- 1. **Immunofluorescence**

Sections of lung tissues were dewaxed and antigenic repair was performed with citric acid. The sections were blocked with 1% goat serum for 1 h at 37 ℃. The Alexa Fluor® 594 anti-mouse F4/80 antibody (BioLegend, San Diego, CA) and Alexa anti-mouse CD4 antibody (Abcam, Cambridge, United Kingdom) were incubated overnight at 4 ℃. Alexa Fluor® 555 anti-rabbit IgG (Abcam, Cambridge, United Kingdom) was incubated for 1 h at 37 ℃. The stained sections were viewed under a fluorescence microscope.

- 1. **Flow Cytometry**

The isolated lung tissues were incubated with 0.5 mg/mL collagenase IV and 8 μg/mL DNase I in HBSS for 35 min at 37 ℃ to obtain a cell suspension. The cell suspension of spleen or lung was passed through a 70 μm cell strainer, and erythrocyte lysate (Solarbio life sciences, Beijing, China) was used to remove erythrocytes. BMDMs and peritoneal cells were washed with cold PBS. Cells were incubated with Fc receptor blocking antibodies (BD Pharmingen) for 10 min at 4 ℃. For cell surface staining, cells were incubated with the following antibodies: anti-F4/80 (APC), anti-CD16/32 (FITC), anti-CD11b (APC), anti-CD24 (PE), anti-MHCII (PerCP/Cyanine5.5), anti-CD45 (PerCP/Cyanine5.5), anti-Siglec-F (PerCP/Cyanine5.5), anti-B220 (APC), anti-CD4 (FITC), anti-Ly6G (PE), and anti-CD11c (FITC), which were purchased from BioLegend (San Diego, CA) , and anti-CD206 (PE), which was purchased from R&D Systems (Minnesota). Next, cells were fixed with 4% formaldehyde for 30 min and perforated with 1% Triton X-100 for 40 min. Intracellular staining was performed using anti-Foxp3 (PE), which were purchased from BD Biosciences. The strained cells were detected with a BD FACSCalibur flow cytometer. The data were analyzed using FlowJo software (Tree Star Inc).

1. **Supplemental Figure**

**Fig. S1. Representative gaiting strategy of BMDMs, lymphocytes in spleen and peritoneal macrophage.**


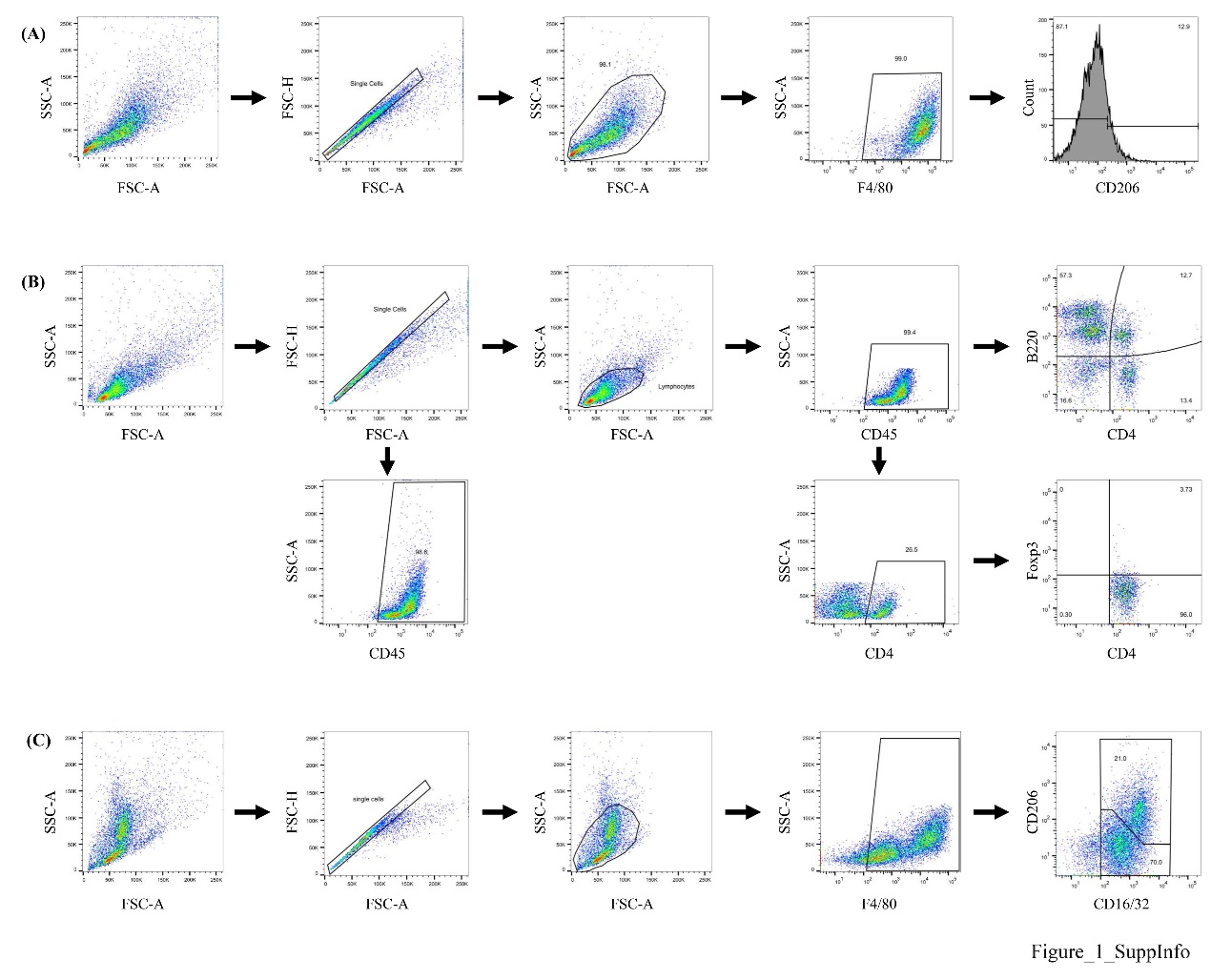


(A) Representative flow cytometry plots for the percentage of F4/80 ^+^ cells and CD206^+^ macrophages in BMDMs were shown. (B) The CD45^+^ immune cells, B220^+^ B cells, CD4^+^ T cells and Foxp3^+^ Treg cells in spleen were identified. (C) The total F4/80^+^ cells and CD16/32^+^ CD206^+^ macrophages in peritoneal cavity were shown.

**Fig. S2. The number of ML in NBL infection group.**


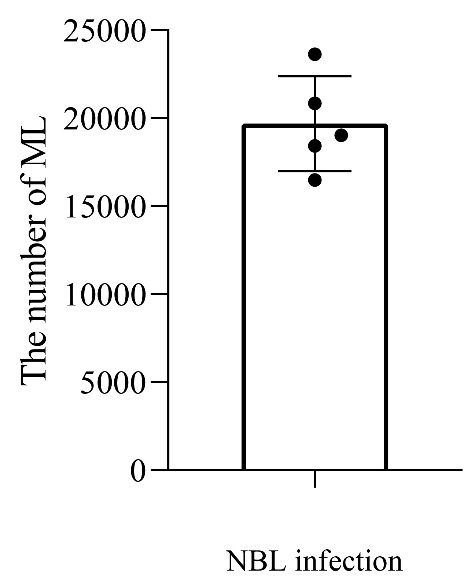


**Fig. S3. Representative gaiting strategy of innate immune cells in lung.**


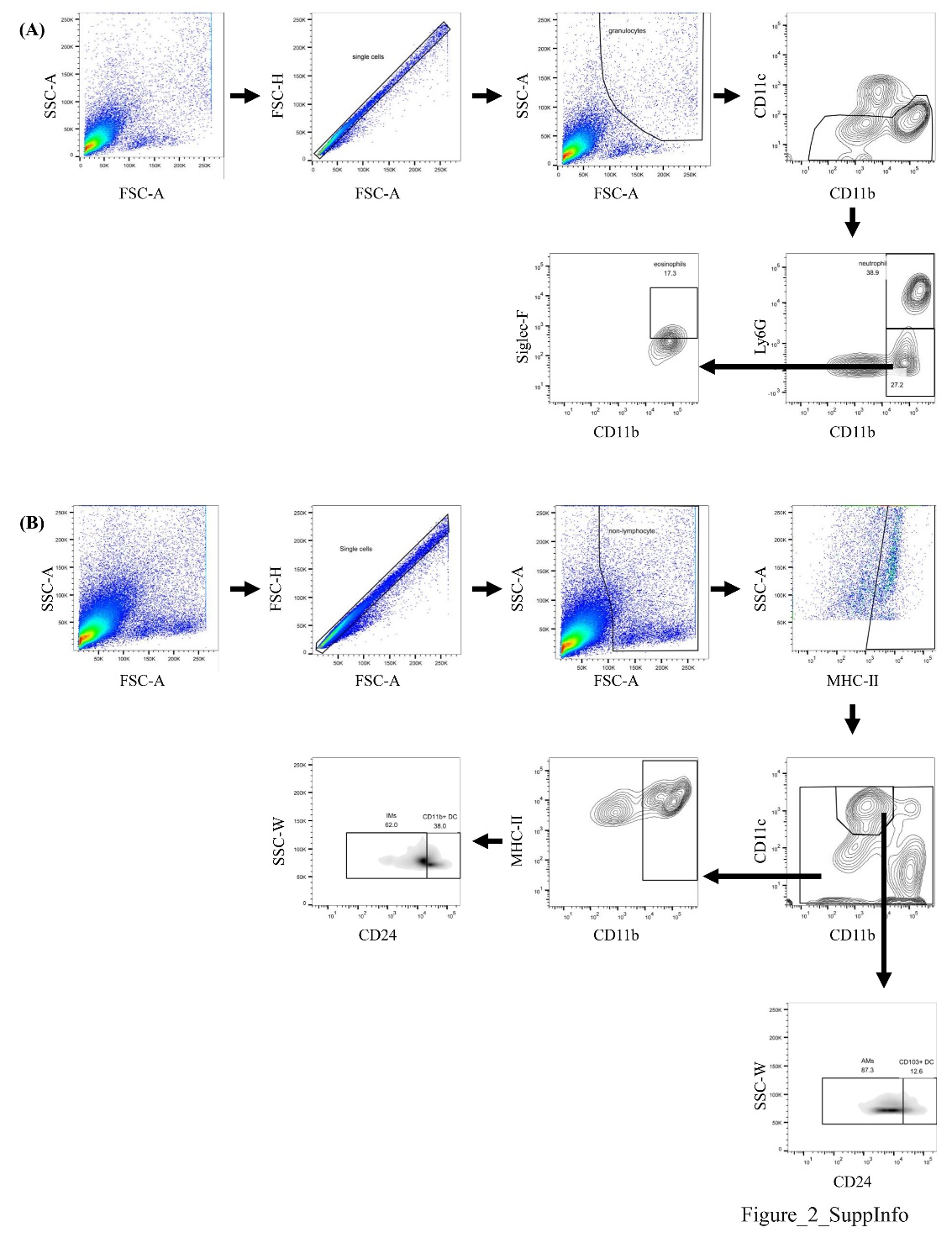


(A) Siglec-F^+^ CD11b^+^ eosinophils and Ly-6G^+^ CD11b^+^ neutrophils were identified. (B) Gaiting strategy of IMs, AMs, CD103^+^ DCs and CD11b^+^ DCs in lung were shown.

1. **Supplemental Tables**

**Table S1. Primer sequences and product size of amplified genes.**

| **Gene name** | **Primer sequence (5’-3’)** | **Product size** |
| --- | --- | --- |
| mIL-25 | F: CGGAGGAGTGGCTGAAGTGGAG | 314bp |
|  | R: ATGGGTACCTTCCTCGCCATG |  |
| mIL-33 | F: ATCACGGCAGAATCATCGAG | 93bp |
|  | R: GCGGTGCTGCTGAACTTT |  |
| mTSLP | F: TTCACTCCCCGACAAAACAT | 105bp |
|  | R: GCCATTTCCTGAGTACCGTC |  |
| mIL-5 | F: GAAGACTTCAGAGTCATGAGAAGGA | 183bp |
|  | R: ATGAGTAGGGACAGGAAGCCT |  |
| mGAPDH | F: ATGACATCAAGAAGGTGGTGAAG | 238bp |
|  | R: TCCTTGGAGGCCATGTAGG |  |
